# The Passing of Murphy's Button

**Published:** 1908-12-26

**Authors:** 


### Surgery.

### THE PASSING OF MURPHY'S BUTTON.

The title of this article refers not as might be expected to the subsequent passage along the alimentary canal of a Murphy's button, which has been used for the purposes of intestinal anastomosis, but to the fact that this ingenious method of uniting intestine has lately fallen into much disuse.

It is not now many years since it was first invented, and the surgeon who devised it is still an active man, but even in this short time it has almost fallen into disuse. It was, when it first came out, as much in advance of the existing methods, such as Senn's bone plates, as its successor, simple suture, is in advance of it. At first it was extensively used in all forms of intestinal anastomosis, but soon arguments were brought forward against it which weighed so strongly with surgeons that it has now fallen into almost complete disuse; particularly when it was pointed out that simple suture had none of these disadvantages.

It was said, for instance, that Murphy's button crushed the layers of intestine contained between its halves, and therefore aimed at obtaining secondary, instead of primary, union. Such a statement may be true, but the argument is of little value if the secondary union obtained is satisfactory, and that is generally the case.

Again, the use of the button necessitates the introduction of a foreign body which is objectionable on principle; and further it may refuse to pass on, and so give rise to intestinal obstruction. The writer can recall a case in which the button was retained for a long period, although it did not give rise to symptoms. The patient, a girl aged twenty-one, complained that she had suffered from abdominal pain for some time. During the last five days the pain had been worse, and had been attended with "vomiting and mucous diarrhœa." She looked flushed and ill. The abdomen was not much distended, and a mass, slightly tender, was felt in the right iliac fossa. The abdomen was opened over the mass. The mesentery was found to be occupied by a number of enlarged glands, tuberculous in nature, and further, the lower end of the ileum and the cæcum were matted together and bound down by dense adhesions. The ileum above this was distended, as if the passage through the mass was obstructed. A lateral anastomosis was made between the distended ileum and the transverse colon, a Murphy's button being used. Convalescence was uneventful, except that six weeks after the operation the button had not been passed, although the patient was getting up daily. A skiagram showed the button behind McBurney's spot on the right side. The abdomen was therefore opened by a median suprapubic incision. The intestine was now healthy, and no free fluid nor enlarged glands were found. The button lay deep down in the right iliac fossa. It was removed by enterotomy, and the patient made an uneventful recovery. The case also illustrates the completeness and the rapidity with which tuberculous peritonitis reacts to simple laparotomy. In this case the button remained *in situ*, but no intestinal obstruction ensued because liquid fæces continued to pass through its lumen.

To counterbalance these disadvantages the button has merits which should recommend it. It often facilitates the actual operation, and it certainly diminishes its duration. We do not imply that Murphy's button is always better than simple suture. In some cases it is manifestly inferior. This is the case in performing a lateral anastomosis generally, but particularly in gastro-jejunostomy. Experience has shown that in the latter operation it does not afford a large enough opening; besides which the button has the whole length of the small and large intestine to travel, even if it does not fall into the stomach and necessitate a subsequent gastrotomy, whereas simple suture is eminently satisfactory in gastro-jejunostomy.

But there are cases in which it is still, according to our view, the best form of intestinal suture. This is the case in end to side anastomosis. The writer recently saw it used with conspicuous success in an operation for excision of the cæcum. The cæcum and some inches of the termination of the ileum were removed. The male portion of the button was then inserted into the ascending colon through its open lower extremity, and its central portion passed from within outwards through a small incision made in the inner wall of the ascending colon. The open lower end of the ascending colon was then closed with a layer of Lembert sutures. The female end was fastened into the end of the ileum with a purse-string suture. The two halves of the button were fixed together, and one reinforcing layer of Lembert sutures was put in round the button. The operation took quite a short time, there was no shock, and the patient made a speedy recovery.

Again, its advantages are great in end-to-end anastomosis of the lower portion of the large intestine; for example, after excision of a carcinoma of the sigmoid. In this position it is not likely to cause any greater constriction of the lumen of the intestine than simple suture; and there is less likelihood of infecting the adjacent peritoneum with the bacillus coli, since no stitches are passed completely through the wall of the bowel, which may form a passage for contaminating material. As in the former case a layer of reinforcing Lembert sutures may be put in to encircle the junction.

In the situations mentioned the button has to pass through large intestine only, and it is not therefore likely to get caught during its transit, although it may conceivably remain at the site of union as in the case quoted above.

Finally the operation of cholecystenterostomy, though rarely called for, is hardly practicable unless a minute Murphy's button is employed.

In view of these considerations it is not correct to suppose that Murphy's button has been entirely superseded by a simple suture. The latter is admittedly excellent, but there are cases in which a Murphy's button is still the most satisfactory and most reliable method of intestinal anastomosis.

